# Relative contributions of personality, psychosocial functioning, and executive cognition to individual differences in delay discounting

**DOI:** 10.3389/fpsyg.2026.1974428

**Published:** 2026-10-21

**Authors:** Yunyan Ye, Zhanyang Liang

**Affiliations:** 1 Institute for Translational Neuroscience and Clinical Psychology, RWTH Aachen University Hospital, Aachen, Germany; 2 Institute of Pathology, RWTH Aachen University Hospital, Aachen, Germany

**Keywords:** delay discounting, executive cognition, human connectome project, incremental prediction, individual differences, personality, psychosocial functioning

## Abstract

**Background:**

Delay discounting (DD) is a widely studied individual-difference phenotype that reflects preferences between smaller immediate and larger delayed rewards. Previous studies have linked DD to demographic characteristics, personality traits, psychosocial functioning, and cognitive abilities. However, the relative incremental contribution of these domains within the same predictive framework remains unclear.

**Methods:**

We analyzed data from 1,193 healthy young adults from the Human Connectome Project–Young Adult dataset. Delay discounting was indexed by the area under the discounting curve for the $200 condition. Participants were randomly divided into a training set (*n* = 953) and a hold-out test set (*n* = 240). Hierarchical models sequentially incorporated demographic variables, personality and psychosocial measures, and executive cognitive measures. Ordinary linear regression, Elastic Net, and XGBoost were evaluated using apparent training performance, nested five-fold cross-validation, and hold-out testing. Model performance was assessed using the coefficient of determination (*R*^2^), Root-mean-square error (RMSE), and Mean Absolute Error (MAE). Incremental value was quantified as the change in *R*^2^ between adjacent models (Δ*R*^2^).

**Results:**

Age and gender alone had little predictive value for DD. In the primary hierarchical models, the largest initial improvement occurred after personality and psychosocial measures were added, with a further increase after cognitive measures were included. The cognition-first analysis, however, showed that cognitive predictors produced similar gains when entered directly after the demographic baseline, particularly with Elastic Net and XGBoost. Thus, the apparent difference between the two predictor domains depended partly on the order in which they were entered. XGBoost fit the training data substantially better than the linear models, but this advantage largely disappeared under cross-validation. In the hold-out test set, Elastic Net performed best (*R*^2^ = 0.0507, RMSE = 0.1976, MAE = 0.1522), although overall prediction remained modest. An expanded sensitivity analysis using 257 available non-imaging predictors likewise showed low validated predictive performance.

**Conclusions:**

Personality-psychosocial and cognitive measures both provided incremental predictive information beyond demographics, although their apparent relative contributions varied with predictor-block order. However, the modest validated performance indicates that delay discounting is influenced by multiple factors beyond those captured by the present models. These findings support a multidomain perspective on individual differences in delay discounting while highlighting the importance of cautious interpretation of predictive performance in large-scale behavioral datasets.

## Introduction

Delay discounting (DD) refers to the decline in the subjective value of a reward as the delay to its receipt increases. Individuals who discount delayed rewards more steeply tend to show a stronger preference for immediate gratification and a weaker tendency to wait for larger future rewards (Odum, 2011a; Myerson et al., 2001; Bailey et al., 2021). Because DD captures meaningful individual differences in preferences for immediate vs. delayed rewards, it has been widely studied in relation to impulsive choice, self-regulation, and everyday behavior (Odum, 2011b). Previous studies have linked steeper DD to addictive behaviors, obesity, gambling disorder, mood disorders, and other conditions characterized by impulsive decision-making, highlighting its relevance across both clinical and non-clinical populations (Kertzman et al., 2022; Mena-Moreno et al., 2022; Miranda-Olivos et al., 2006; Strickland et al., 2021).

Individual differences in DD have been associated with a wide range of demographic, psychological, and cognitive characteristics. Sociodemographic characteristics may capture differences in life stage and socioeconomic context that shape how individuals evaluate immediate and delayed rewards, although associations with discounting behavior, such as those involving age and socioeconomic conditions, are generally modest and sometimes inconsistent across populations (Downey et al., 2022; Seaman et al., 2022; Wan et al., 2024). Personality traits are relatively stable across situations and have been examined as potential correlates of intertemporal choice (Yeh et al., 2021; Roberts and Yoon, 2022). For example, conscientiousness may be related to self-regulation and future-oriented behavior, whereas neuroticism and extraversion may be related to affective and reward-related tendencies. Although self-report measures of personality and behavioral measures often show only weak correlations (Dang et al., 2020), previous studies have likewise found only weak associations between DD and psychometric measures of impulsivity or broader personality traits (Yeh et al., 2021; MacKillop et al., 2016). This may partly reflect the fact that these measures capture different aspects of behavior. Psychosocial characteristics have also been examined in relation to DD, including perceived stress and emotional functioning, although the strength and direction of these associations have varied across studies (Yeh et al., 2021; DeAngelis et al., 2022). Intertemporal choice places demands on cognitive processes involved in representing future outcomes, comparing rewards across time, and regulating responses to immediate incentives. Executive functions, particularly working memory and inhibitory control, may therefore be relevant to individual differences in DD. However, reported associations are generally small and vary across cognitive measures and samples (Yeh et al., 2021; Wesley and Bickel, 2013). Overall, existing evidence indicates that DD is associated with multiple domains of individual differences rather than a single psychological construct.

DD is sensitive to situational and experimental conditions, whereas personality traits are generally conceptualized as relatively stable individual characteristics (Odum, 2011a; Odum et al., 2020). These different properties do not necessarily make their association uninformative. Rather, examining personality in relation to DD can show whether relatively stable traits account for some between-person differences in discounting. More broadly, DD may show systematic between-person differences while remaining sensitive to situational and experimental conditions (Odum, 2011b; Odum et al., 2020). Associations with sociodemographic, personality, psychosocial, and cognitive characteristics therefore need not imply that these characteristics determine discounting or that DD is fixed across contexts.

Despite the growing literature, an important question remains unresolved. Previous studies have commonly examined associations between DD and selected demographic variables, personality traits, psychosocial measures, or cognitive abilities. Fewer studies have directly compared the incremental contribution of these domains within the same analytical framework. Consequently, it remains unclear whether these domains provide complementary predictive information or whether their associations with DD largely reflect shared variance. Assessing these domains in the same model may clarify whether they make distinct or overlapping contributions to individual differences in DD.

The availability of large, deeply phenotyped datasets provides an opportunity to address this question more systematically. Such datasets allow us to compare how much different domains contribute to the prediction of DD while evaluating whether these patterns are consistent across different modeling approaches and validation strategies.

The present study examined the relative incremental contributions of demographic characteristics, personality and psychosocial measures, and executive cognitive measures to individual differences in delay discounting in the Human Connectome Project–Young Adult (HCP-YA) dataset. Rather than developing the most accurate prediction model, our primary aim was to determine whether the relative contribution of these predictor domains remained consistent across different modeling approaches.

## Materials

### Participants and procedure

This study used individual-difference and behavioral data from the HCP-YA dataset, obtained through ConnectomeDB platform (https://www.humanconnectome.org/study/hcp-young-adult/data-releases). The initial sample comprised 1,206 healthy young adults aged 22–35 years. Participants with missing data on any study variable were excluded, leaving a final analytic sample of 1,193 participants. Using a fixed random seed, the sample was divided into a training set (*n* = 953; 80%) and a hold-out test set (*n* = 240; 20%), balancing model development with independent hold-out evaluation.

### Measures

Except for gender and age, all HCP-YA questionnaire variables and task-based measures used in this study were based on the unadjusted raw scores provided in the database. Age-adjusted scores or other standardized scores were not used. This choice was intended to preserve between-person variability and avoid removing age-related variance before modeling, as age was included explicitly in the demographic baseline model.

The HCP-YA assessed delay discounting using an adjusting-amount procedure in which participants chose between a smaller immediate reward and a larger delayed reward (Du et al., 2002). The immediate amount was adjusted across choices to estimate indifference points at different delays, without assuming a specific mathematical form of the discounting function.

#### Outcome variable

The outcome variable was DD area under the curve for $200(DDisc_AUC_200). This AUC score is a relatively neutral, non-parametric index calculated from the empirical discounting curve (Myerson et al., 2001). AUC values range from 0 to 1, with higher values indicating shallower discounting and a weaker preference for immediate rewards, whereas lower values indicate steeper discounting and a stronger preference for immediate rewards.

#### Covariates

Covariates were grouped into three domains. The demographic domain served as the baseline covariate domain and included age and gender. The personality-psychosocial domain included both personality traits and psychosocial functioning. Personality traits were assessed using the NEO Five-Factor Inventory and included Agreeableness, Openness, Conscientiousness, Neuroticism, and Extraversion. Psychosocial functioning was assessed using the NIH Toolbox General Life Satisfaction Survey, Meaning and Purpose Survey, Positive Affect Survey, Friendship Survey, Loneliness Survey, Perceived Stress Survey, and Self-Efficacy Survey. Personality traits capture relatively stable individual differences, whereas psychosocial variables reflect adaptation in emotional experience, perceived stress, self-efficacy, social relationships, life satisfaction, and goal maintenance. Therefore, personality traits and psychosocial functioning were entered into the same model layer to evaluate their shared contribution to individual-difference information (Roberts and Yoon, 2022). Executive cognitive measures included the Penn Progressive Matrices: number of Correct Responses, NIH Toolbox Pattern Comparison Processing Speed Test, NIH Toolbox List Sorting Working Memory Test, NIH Toolbox Flanker Inhibitory Control and Attention Test, NIH Toolbox Dimensional Change Card Sort Test, and Short Penn Continuous Performance Test: true Positives. The primary predictor set was defined conceptually to represent the domains targeted by the study question rather than selected through data-driven screening of the full HCP-YA phenotypic battery. Predictor inclusion was not based on the strength of association with delay discounting; detailed selection rationale is provided in Supplementary Table S1.

#### Analytic approach

All statistical analyses were performed using R software version 4.5.3. The training set was used for model development, hyperparameter tuning, and internal cross-validation. The hold-out test set was used to evaluate model performance in participants who were not included in model training. Notably, the hold-out test set was drawn from the same HCP-YA sample. This step should be interpreted as within-dataset hold-out evaluation rather than external validation.

Three hierarchical models were constructed to evaluate the incremental contribution of each predictor domain. Model 0 included age and gender and served as a baseline reference for evaluating the incremental contribution of the additional predictor domains. Model 1 added personality traits and psychosocial functioning to Model 0. Model 2 further added neurocognitive variables to Model 1. This structure allowed us to assess whether each added domain provided incremental predictive value beyond the variables already included in the previous model. To assess sensitivity to predictor-block order, an additional cognition-first model Model 1 (B) was fitted by adding the cognitive domain directly to Model 0, whereas the original personality-psychosocial model was denoted Model 1. Model 1 and Model 1 (B) therefore represented parallel extensions of the same demographic baseline and were evaluated in the same training participants using identical outer cross-validation folds.

For each stage of the hierarchical modeling framework, we fitted three predictive models: ordinary linear regression, Elastic Net regularization (Elastic Net) (Friedman et al., 2010; Tay et al., 2023), and eXtreme Gradient Boosting (XGBoost) (Chen and Guestrin, 2016). Ordinary linear regression provided a conventional linear benchmark. Elastic Net is a regularized linear approach that can reduce overfitting and handle correlated predictors. XGBoost is a tree-based non-linear method that can capture more complex associations and interactions. These methods were selected to assess whether the incremental contribution of each predictor domain was consistent across different modeling approaches. For Elastic Net, predictors were standardized internally during model fitting. Predictor scaling was not required for ordinary linear regression or tree-based XGBoost. Because flexible models can produce overly optimistic results when evaluated only in the training set, we assessed model performance using five-fold cross-validation (five-fold CV) and hold-out test-set evaluation. This design allowed us to distinguish apparent model fit from more stable predictive performance within the same dataset. However, the goal was not to identify the most complex or best-fitting algorithm.

Model performance was assessed using *R*^2^, Root-mean-square error (RMSE), and Mean Absolute Error (MAE). Higher *R*^2^ values indicate that a greater proportion of variance is explained by the model, whereas lower RMSE and MAE values indicate smaller prediction errors and better predictive performance. Incremental value was quantified as the change in *R*^2^ between adjacent hierarchical models, with Δ*R*^2^ defined as the *R*^2^ of the subsequent hierarchical model minus that of the previous model. We also examined changes in RMSE and MAE across models. Apparent training set performance, nested cross-validation, and hold-out test-set evaluation were interpreted together to assess whether each added predictor domain provided stable incremental predictive value. The analytic workflow is presented in Figure 1.

**Figure 1:**
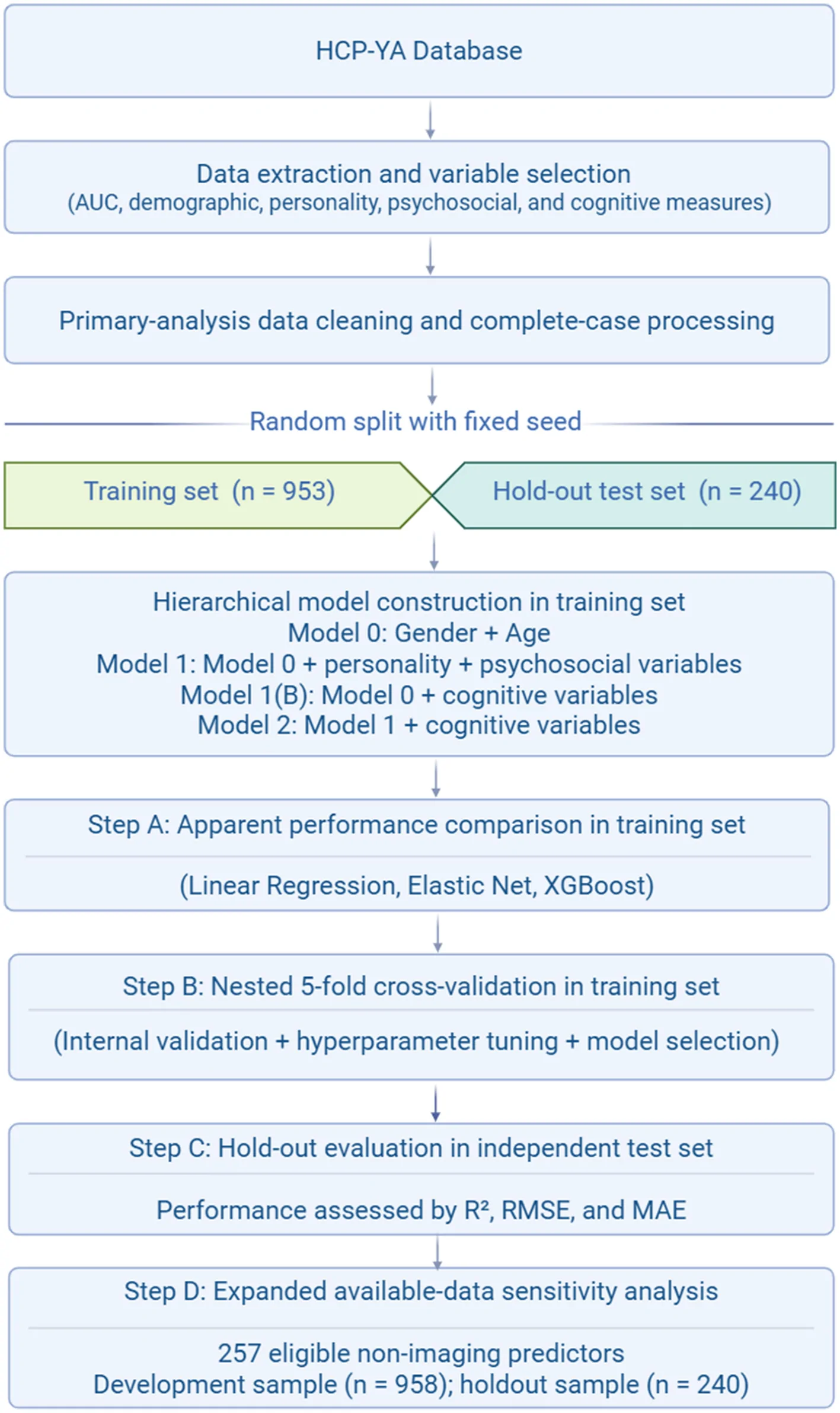
Statistical analysis workflow.

#### Expanded available-data sensitivity analysis

To assess whether the conceptually defined primary predictor set omitted potentially informative measures available elsewhere in the HCP-YA phenotypic dataset, we conducted an expanded sensitivity analysis using all eligible non-imaging measures available under our current data-access authorization. This analysis was reconstructed from the original HCP-YA export of 1,206 participants. After excluding imaging-derived, administrative/technical, age-adjusted duplicate, and delay-discounting-derived variables, 268 candidate predictors were identified. Eight participants with missing outcome data were excluded, leaving 1,198 participants. Five Social Task conditional reaction-time variables with >50% missingness and six invariant variables were subsequently removed, yielding 257 predictors; no retained predictor had >20% missingness. Missing predictor values were handled using training-fold-specific imputation, with continuous variables imputed by the training-partition median and categorical variables by the training-partition mode, and the resulting values applied unchanged to the corresponding validation partition. Participants were divided into a development sample (*n* = 958) and an independent holdout sample (*n* = 240). Model development used nested five-fold cross-validation with imputation embedded within the resampling procedure. For Elastic Net, α was tuned from 0 to 1 in increments of 0.1, with λ selected by inner-validation performance; XGBoost used the same nested cross-validation framework and hyperparameter search strategy as in the primary analysis. Final models were refitted using all 958 development participants and evaluated once in the untouched 240-participant holdout sample. Variable importance was assessed separately for the two modeling approaches. For the Elastic Net framework, predictor importance was summarized using standardized coefficient magnitudes; for multi-level categorical predictors, importance was aggregated across the corresponding encoded terms using the Euclidean norm. For XGBoost, importance was quantified in the independent holdout sample using mean absolute SHapley Additive exPlanations (SHAP) values, with larger values indicating greater average contributions to model predictions (Lundberg et al., 2020).

## Results

### Apparent performance in the training set

As shown in Table 1, Model 0 showed weak apparent predictive performance and limited predictive ability across all three analytical methods. This indicates that age and gender alone had limited ability to predict DD. After personality traits and psychosocial functioning were added, Model 1 showed the largest improvement in performance. Compared with Model 0, the Δ*R*^2^ values for Model 1 were 0.0599, 0.0599, and 0.4809 for linear regression, Elastic Net, and XGBoost, respectively. RMSE and MAE also decreased across the three methods. These results suggest that, in the apparent training-set analysis, the personality-psychosocial domain provided the largest incremental increase for predicting delay discounting. After neurocognitive variables were further added, Model 2 showed a further increase, but the magnitude of improvement was smaller than that observed from Model 0 to Model 1. Compared with Model 1, the Δ*R*^2^ values for Model 2 were 0.0172, 0.0172, and 0.0442 across the three methods. This indicates that neurocognitive variables provided further but more modest supplementary information at the apparent-performance level. Linear regression and Elastic Net showed very similar apparent performance. In contrast, XGBoost showed stronger apparent fit in the training set, with the highest apparent *R*^2^ observed in Model 2 (*R*^2^ = 0.5344). However, this high apparent fit should be interpreted together with the subsequent nested cross-validation and hold-out test-set results to determine whether it reflects stable predictive signal rather than optimism introduced by model flexibility.

**Table 1:** Apparent performance of different models and algorithms in the training set.

Model	Method	*R* ^2^	RMSE	MAE
Model 0	Linear regression	0.0076	0.2046	0.1579
	Elastic net	0.0076	0.2046	0.1579
	XGBoost	0.0092	0.2044	0.1576
Model 1	Linear regression	0.0676	0.1983	0.1511
	Elastic net	0.0676	0.1983	0.1511
	XGBoost	0.4901	0.1467	0.1131
Model 2	Linear regression	0.0847	0.1965	0.1501
	Elastic net	0.0847	0.1965	0.1501
	XGBoost	0.5344	0.1401	0.1088

### Five-fold cross-validation and hyperparameter tuning in the training set

Five-fold CV was conducted within the training set for hyperparameter tuning and internal validation. Model 0 performed poorly across all three methods, with *R*^2^ values below zero. This indicates that age and gender alone provided almost no cross-validated predictive value for delay discounting. Adding personality traits and psychosocial functioning produced small increases in performance across methods. The Δ*R*^2^ values from Model 0 to Model 1 were 0.0282 for linear regression, 0.0321 for Elastic Net, and 0.0389 for XGBoost. Prediction errors also decreased, indicating a limited incremental contribution from the personality-psychosocial domain. Adding neurocognitive variables produced a further but smaller improvement. The Δ*R*^2^ values from Model 1 to Model 2 were 0.0031, 0.0203, and 0.0151 across the three methods. Thus, neurocognitive variables added only limited additional predictive value beyond the personality-psychosocial domain (Table 2).

**Table 2:** Predictive performance of each hierarchical model under nested five-fold cross-validation.

Model	Method	*R* ^2^	RMSE	MAE
Model 0	Linear regression	−0.0037	0.2058	0.1587
	Elastic net	−0.0029	0.2057	0.1585
	XGBoost	−0.0015	0.2055	0.1586
Model 1	Linear regression	0.0245	0.2029	0.1548
	Elastic net	0.0292	0.2024	0.1548
	XGBoost	0.0373	0.2015	0.1548
Model 2	Linear regression	0.0275	0.2025	0.1550
	Elastic net	0.0495	0.2002	0.1531
	XGBoost	0.0524	0.1999	0.1535

In the cognition-first sensitivity analysis, Model 1 (B) yielded cross-validated *R*^2^ values of 0.0173, 0.0249, and 0.0366 for linear regression, Elastic Net, and XGBoost, with corresponding Δ*R*^2^ values of 0.0210, 0.0278, and 0.0440 relative to Model 0. These increments were broadly comparable to those for Model 1, particularly for Elastic Net and XGBoost, suggesting that the relative contribution of the two predictor domains partly depended on block-entry order.

Based on the lowest RMSE, Model 2 with XGBoost showed the highest cross-validated performance relative to the other models (*R*^2^ = 0.0524, RMSE = 0.1999, MAE = 0.1535), followed closely by Model 2 with Elastic Net (*R*^2^ = 0.0495, RMSE = 0.2002, MAE = 0.1531). The difference between these two models was small. Notably, the strong apparent advantage of XGBoost was greatly attenuated under nested cross-validation. Its *R*^2^ in Model 2 decreased from 0.5344 in the apparent training-set analysis to 0.0524 under cross-validation, indicating that much of its training-set advantage likely reflected model flexibility rather than robust predictive signal.

Figure 2 shows the prediction distribution of Model 2 under nested cross-validation. Overall, the model showed limited ability to identify individuals with extremely low or extremely high AUC values, with predicted values clearly shrinking toward the mean. Many individuals with very low observed AUC values, approximately 0.02–0.08, were still predicted within the range of 0.15–0.30. Similarly, many individuals with very high observed AUC values, approximately 0.80–0.98, had predicted values concentrated around 0.25–0.40. Thus, the main advantage of the fuller predictor set was a modest reduction in overall prediction error, rather than accurate identification of extreme DD profiles at the individual level.

**Figure 2:**
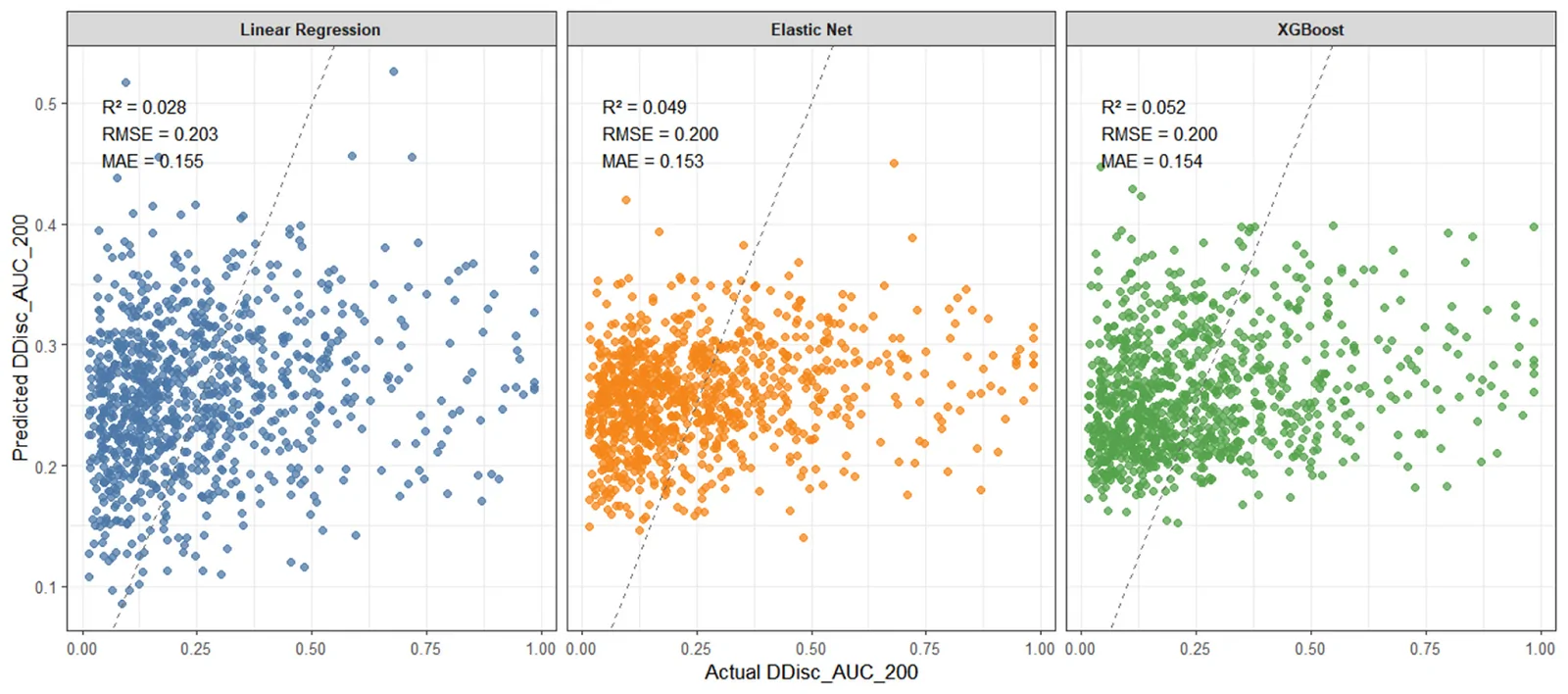
Prediction distribution of AUC under cross-validation.

### Hold-out test set evaluation

Table 3 presents the performance of Model 2 in the hold-out test set (*n* = 240). The final models used the hyperparameters selected during cross-validation in the training set (Supplementary Table S2). Elastic Net showed the most stable hold-out performance, whereas XGBoost did not maintain its strong apparent training-set advantage. Although overall predictive accuracy remained low, the hold-out results still supported a weak but measurable incremental signal from the personality–psychosocial and neurocognitive domains.

**Table 3:** Predictive performance of Model 2 in the hold-out test set.

Method	*R* ^2^	RMSE	MAE
Linear regression	0.0477	0.1979	0.1531
Elastic net	0.0507	0.1976	0.1522
XGBoost	0.0331	0.1994	0.1544

### Expanded available-data sensitivity analysis

In the expanded available-data sensitivity analysis, 257 eligible non-imaging predictors were evaluated in 1,198 participants. Despite the substantially broader predictor set, predictive performance remained modest under both nested cross-validation and independent holdout evaluation, with Elastic Net showing somewhat better performance than XGBoost (Supplementary Table S3). Variable-importance analyses showed broadly consistent rankings across the two modeling approaches. Pittsburgh Sleep Quality Index amount of sleep, the NIH Toolbox Cognition Crystallized Composite, and the NIH Toolbox Picture Vocabulary Test were the three highest-ranking predictors in both models, and 11 of the top 20 predictors overlapped between Elastic Net and XGBoost. The highest-ranking predictors spanned sleep, cognition, personality, psychosocial functioning, and task-based behavioral measures (Supplementary Table S4).

## Discussion

The present study compared the relative incremental predictive information provided by age and gender, personality and psychosocial measures, and executive cognitive measures for individual differences in delay discounting in a large sample of healthy young adults. In the primary hierarchical sequence, personality and psychosocial measures produced the larger initial increment beyond the age-and-gender baseline, whereas the cognition-first sensitivity analysis indicated that the relative contributions of the two domains partly depended on block-entry order. However, the overall predictive performance remained modest across validation procedures, indicating that the majority of variance in delay discounting was not explained by the predictor domains examined here.

Another notable finding was the consistent distinction between apparent training performance and validated predictive performance. Although XGBoost achieved substantially higher apparent fit in the training data, this advantage was markedly attenuated under nested cross-validation and was not maintained in the hold-out sample. By contrast, Elastic Net showed more stable training performance across cross-validation and hold-out testing despite lower apparent training fit, highlighting the importance of evaluating predictive models using validation procedures rather than relying solely on in-sample performance.

The expanded available-data sensitivity analysis further supported this interpretation. When the predictor space was broadened from the conceptually defined primary predictor set to 257 available non-imaging measures, validated performance remained low. At the same time, substantial overlap among higher-ranking predictors across models, including several variables from or closely related to the primary predictor set, indicated some consistency between the original domain-based specification and the broader data-driven analysis. Taken together, these findings suggest that the low predictive performance observed in the primary analyses cannot be attributed solely to the relatively restricted predictor set.

### Interpretation of domain-level contributions

#### Personality and psychosocial measures

In the primary hierarchical sequence, the combined personality and psychosocial module provided a larger initial increment beyond the age-and-gender baseline. One important consideration is that the incremental variance attributed to a predictor block in hierarchical regression depends on the order in which blocks are entered. When correlated predictor domains share explanatory variance, the block entered earlier may absorb variance that could otherwise be attributed to a later block. Accordingly, the change in *R*^2^ associated with each predictor block represents its additional contribution conditional on the predictors already entered, rather than an order-independent estimate of its relative importance. More comprehensive assessments of predictor importance generally evaluate contributions across multiple possible predictor combinations rather than relying on a single prespecified sequence (Johnson and LeBreton, 2004; Mizumoto, 2023). Because the personality and psychosocial module preceded the executive cognitive module in the present analysis, some variance shared between these domains may have been assigned to the earlier block. The larger increment associated with the personality and psychosocial module should therefore not be interpreted as demonstrating that personality and psychosocial characteristics are intrinsically more important determinants of delay discounting than executive cognition.

Importantly, the cognition-first sensitivity analysis indicated that the apparent relative contribution of the two domains was partly influenced by block-entry order. Nevertheless, these findings do not establish that personality and psychosocial characteristics are inherently more important than executive cognition, because the apparent relative contributions of the two domains were partly influenced by block-entry order and shared variance among predictors. One possible explanation is that the personality and psychosocial module sampled a broader range of between-person characteristics than the executive cognitive module. The former combined dispositional traits with measures of current psychological and social functioning, whereas the latter consisted of a more restricted set of laboratory-based executive tasks. Its larger predictive increment may therefore reflect broader construct coverage, differences in measurement reliability, or both, rather than a clear hierarchy between personality and cognition. This distinction is important because predictive performance and explanatory or causal importance are not equivalent: a domain may improve prediction without being the dominant causal influence on the outcome (Shmueli, 2010; Yarkoni and Westfall, 2017).

#### Executive cognitive measures

When entered after personality and psychosocial variables in the primary hierarchical sequence, cognitive measures provided a relatively smaller additional increment. However, the cognition-first sensitivity analysis showed that cognitive predictors produced broadly comparable improvements when entered directly after the demographic baseline, particularly under Elastic Net and XGBoost. Thus, the smaller increment observed in the primary hierarchy should be interpreted as the additional contribution of cognition conditional on the previously entered personality-psychosocial variables, rather than as evidence of generally weaker predictive relevance.

The limited predictive contribution of the cognitive measures should not be attributed only to missing cognitive variables. In the cognition-first analysis, cognitive predictors showed gains similar to those of the personality-psychosocial domain when each was added after the demographic baseline. This suggests that the smaller cognitive increment in the original model was partly related to block-entry order. DD is also sensitive to situational and experimental conditions, which may limit how much stable individual characteristics can explain. Intertemporal choices are also shaped by reward valuation, motivational states, affective processes, and contextual factors (Bailey et al., 2021; Chen et al., 2021). In addition, laboratory-based cognitive tasks may have limited reliability for individual-difference research (Hedge et al., 2018; Kucina et al., 2023), and reliability can vary across tasks and task designs (Kadlec et al., 2024; Schuch et al., 2022). The available cognitive measures also did not cover all relevant processes, such as episodic future thinking (Yeh et al., 2021; Ye et al., 2022; Cona et al., 2023). Together, these factors may help explain the limited predictive performance.

### Overall prediction still modest

Although predictor domains differed in their relative contributions, the overall predictive performance remained modest across all validation procedures. Even the best-performing model explained only a limited proportion of the variance in delay discounting, indicating that substantial individual variability remained unexplained.

The low predictive performance may reflect the experimental procedure and conditions under which DD was measured. Individual variability can depend partly on experimental control and the procedures used to obtain behavioral data (Branch, 2021). Because DD was assessed using a single standardized HCP-YA procedure, we could not examine whether individual differences would vary across different task procedures or experimental conditions. DD may also vary across time and context (Miller et al., 2023). In addition, the relatively narrow age range and generally good health of the HCP young-adult sample may have restricted variability in both the predictors and the outcome.

The present findings are broadly consistent with previous studies showing that delay discounting is associated with multiple domains, although their predictive contributions in the present study were small. However, most previous studies have focused on individual predictor domains rather than evaluating multiple domains within the same analytical framework, making direct comparisons of their relative contributions difficult (Bailey et al., 2021; Keidel et al., 2021). By evaluating demographic, personality, psychosocial, and executive cognitive variables within the same analytical framework, the present study provides a more balanced assessment of their relative predictive contributions while accounting for overlap among predictors.

The findings suggest that personality, psychosocial, and executive cognitive measures provide complementary rather than competing information about delay discounting. The modest predictive performance across models suggests that delay discounting is unlikely to be adequately predicted by any single domain. Future studies may need to consider a wider range of behavioral, environmental, developmental, and biological factors.

### Methodological implications

Beyond the substantive findings, the present study also has several methodological implications for research on individual differences in delay discounting. Previous studies have often focused on identifying statistically significant associations between individual predictors and delay discounting using conventional regression analyses. Although such studies identify variables associated with delay discounting, they provide limited evidence about whether these associations can predict delay discounting in previously unseen individuals (Shmueli, 2010; Yarkoni and Westfall, 2017). They also do not show whether a variable adds predictive value once other correlated measures are considered.

The present study therefore adopted a comparative modeling framework that evaluated ordinary linear regression, Elastic Net, and XGBoost using apparent training performance, nested cross-validation, and hold-out testing. This design enabled comparison not only of model fit but also of model generalizability across increasingly stringent validation procedures. Although XGBoost achieved substantially higher apparent training performance, this advantage decreased markedly under nested cross-validation and was not maintained in the hold-out sample. By contrast, Elastic Net produced comparatively stable performance across validation procedures despite lower apparent training fit. These findings suggest that model ranking depends strongly on the validation strategy adopted. The apparent superiority of XGBoost disappeared after increasingly stringent validation, whereas Elastic Net showed more stable performance across samples.

Beyond the comparison of modeling approaches, the present results also illustrate that statistically significant associations do not necessarily translate into strong out-of-sample prediction (Hofman et al., 2021; Rosenbusch et al., 2019). Even when multiple theoretically relevant domains were analyzed simultaneously, prediction remained modest in the hold-out test set. By evaluating multiple predictor domains within the same analytical framework and evaluating the models in held-out participants, the present study showed that variables explaining individual differences are not necessarily those that provide robust out-of-sample prediction. This distinction underscores the importance of evaluating predictive performance in a hold-out test set rather than relying solely on statistical significance (Hofman et al., 2021). Notably, a separate hold-out test set is most appropriate when the sample is sufficiently large and an independent evaluation of final model performance is required.

### Strengths and limitations

The present study has several strengths. It used a relatively large HCP-YA sample with measure spanning personality, psychosocial functioning, and cognition, allowing multiple predictor domains to be evaluated within the same analytical framework. The combination of ordinary linear regression, Elastic Net, and XGBoost, together with nested cross-validation and within-dataset hold-out testing, also provided a more rigorous assessment of predictive performance than reliance on in-sample model fit alone. The expanded available-data sensitivity analysis further examined whether the findings were sensitive to the relatively restricted, conceptually defined primary predictor set.

Several limitations should be considered. The cross-sectional design does not permit causal or temporal conclusions, and the restriction to healthy young adults may limit generalizability to other age groups and clinical populations. In addition, DD was assessed using a single standardized procedure, so we could not examine whether individual differences would vary across different task procedures or experimental conditions. Finally, predictive performance remained low, indicating that substantial individual variability in DD was not captured by the present models. Although the expanded sensitivity analysis substantially broadened predictor coverage, it was limited to eligible non-imaging measures available under our current HCP-YA data-access authorization. Measures from the Health and Family History, Psychiatric and Life Function, and Substance Use domains were not available within the dataset accessible under this authorization and were not included in the expanded analysis. Accordingly, this analysis should not be interpreted as an exhaustive assessment of all potentially relevant HCP-YA phenotypic measures.

## Conclusions

Personality-psychosocial and cognitive measures both provided small incremental predictive gains beyond demographics, although their apparent relative contributions varied with predictor-block order. Nevertheless, performance remained low in the hold-out test set, indicating that the available measures captured only a limited proportion of individual differences in delay discounting. These findings support a multidomain account of delay discounting and highlight the need for broader predictors and rigorous out-of-sample validation in future research.

## Data Availability

The data can be found here: human Connectome Project: Young Adult (HCP-YA), ConnectomeDB: https://db.humanconnectome.org. Data access requires registration and agreement to the applicable HCP data use terms. No accession number is applicable. Further inquiries can be directed to the corresponding author.
