# Supplementary material for: Relative contributions of personality, psychosocial functioning, and executive cognition to individual differences in delay discounting
Source: Front Psychol. 2026 Oct 21;17:1974428. doi: 10.3389/fpsyg.2026.1974428 (PMC13731279; doi:10.3389/fpsyg.2026.1974428)
Supplement: Supplementary file 1 [file Supplementary_file_1.pdf]

Supplementary Material

Supplementary Table S1. Predictor domains and variables included in each hierarchical model

Model	Predictor domain(s)	Predictors
Model 0	Demographic	Age; Gender
Model 1	Demographic and personality and psychosocial	Age; Gender; Agreeableness; Openness; Conscientiousness; Neuroticism; Extraversion; General Life Satisfaction; Meaning and Purpose; Positive Affect; Friendship; Loneliness; Perceived Stress; Self-Efficacy
Model 1(B)	Demographic and cognition	Age; Gender; Penn Progressive Matrices: Number of Correct Responses; NIH Toolbox Pattern Comparison Processing Speed Test; NIH Toolbox List Sorting Working Memory Test; NIH Toolbox Flanker Inhibitory Control and Attention Test; NIH Toolbox Dimensional Change Card Sort Test; Short Penn Continuous Performance Test: True Positives
Model 2	Demographic and personality and psychosocial and cognition	All predictors included in Model 1 plus all cognitive predictors included in Model 1(B)

Note. The demographic model was intentionally restricted to age and gender as a minimal reference model. For personality, all five higher-order NEO Five-Factor Inventory domain scores were included, whereas the individual questionnaire items were not entered separately. The psychosocial set was defined to represent psychological well-being, companionship-related social relationships, perceived stress, and self-efficacy; measures from distinct emotion or social-functioning subdomains were not included in the primary construct definition. The executive cognition set was selected to represent executive and related cognitive functions relevant to controlled decision-making; age-adjusted duplicates, delay-discounting-derived measures, secondary or redundant task indices, and overlapping composite scores were not included. Predictor inclusion was not based on the strength of association with delay discounting.

Supplementary Table S2. Selected hyperparameters for Model 2 after nested five-fold cross-validation

Method	Hyperparameter	Selected value
Elastic Net	mixing parameter (alpha)	0.5
Elastic Net	regularization parameter (lambda)	0.0132
XGBoost	learning rate (eta)	0.03
XGBoost	maximum tree depth	3
XGBoost	minimum child weight	1
XGBoost	number of boosting rounds (nrounds)	94

Supplementary Table S3. Predictive performance of the expanded available-data sensitivity analysis

Validation procedure	Method	N	R^2	RMSE	MAE
nested five-fold cross-validation	Elastic Net	958	0.0910	0.1966	0.1514
hold-out test	Elastic Net	240	0.0985	0.1880	0.1404
nested five-fold cross-validation	XGBoost	958	0.0680	0.1990	0.1525
hold-out test	XGBoost	240	0.0634	0.1917	0.1415

Note: The expanded sensitivity analysis initially considered 268 eligible non-imaging predictors. Five Social Cognition Task conditional response-time measures were excluded because more than 50% of observations were missing: median response time for a Random response during Theory-of-Mind trials;

median response time for an Unsure response during Theory-of-Mind trials; median response time for a Theory-of-Mind response during Random trials; median response time for an Unsure response during Random trials; and overall median response time for Unsure responses. Six additional variables were excluded because they showed no between-participant variation: percentage of no-response trials in the Gambling Task; percentage of no-response trials in reward blocks of the Gambling Task; percentage of no-response trials in punishment blocks of the Gambling Task; percentage of no-response trials in the Social Cognition Task; percentage of no-response trials in Random blocks of the Social Cognition Task; and percentage of no-response trials in Theory-of-Mind blocks of the Social Cognition Task. The final expanded predictor set therefore comprised 257 variables. Missing values in retained predictors were handled using training-fold-specific median imputation for continuous variables and mode imputation for categorical variables.

Supplementary Table S4. Highest-ranking predictors in the expanded available-data sensitivity analysis

Rank	Elastic Net	|β|	XGBoost	Mean absolute SHAP
1	Pittsburgh Sleep Quality Index: Amount of Sleep	0.0350	Pittsburgh Sleep Quality Index: Amount of Sleep	0.0092
2	NIH Toolbox Cognition Crystallized Composite: Unadjusted Scale Score	0.0267	NIH Toolbox Cognition Crystallized Composite: Unadjusted Scale Score	0.0081
3	NIH Toolbox Picture Vocabulary Test: Unadjusted Scale Score	0.0265	NIH Toolbox Picture Vocabulary Test: Unadjusted Scale Score	0.0069
4	Age	0.0234	NEO Five-Factor Inventory Item 29	0.0068
5	Pittsburgh Sleep Quality Index Component 3: Sleep Duration	0.0233	NEO Five-Factor Inventory: Openness to Experience	0.0058
6	NEO Five-Factor Inventory Item 29	0.0231	NIH Toolbox 2-Minute Walk Endurance Test: Unadjusted Scale Score	0.0051
7	NEO Five-Factor Inventory Item 24	0.0223	NEO Five-Factor Inventory Item 13	0.0043
8	Relational Processing Task: Median Response Time for Match Trials	0.0222	Penn Progressive Matrices: Median Reaction Time for Correct Responses	0.0042
9	NEO Five-Factor Inventory Item 13	0.0215	NIH Toolbox Oral Reading Recognition Test: Unadjusted Scale Score	0.0032
10	NIH Toolbox Oral Reading Recognition Test: Unadjusted Scale Score	0.0204	Penn Progressive Matrices: Number of Correct Responses	0.0031
11	NEO Five-Factor Inventory Item 47	0.0198	Relational Processing Task: Median Response Time for Match Trials	0.0029
12	NIH Toolbox 2-Minute Walk Endurance Test: Unadjusted Scale Score	0.0195	NIH Toolbox Anger-Hostility Survey: Unadjusted Scale Score	0.0024
13	NEO Five-Factor Inventory Item 18	0.0189	Pittsburgh Sleep Quality Index Component 3: Sleep Duration	0.0023
14	Pittsburgh Sleep Quality Index Component 4: Habitual Sleep	0.0186	Pittsburgh Sleep Quality Index: Global Score	0.0023

	Efficiency			
15	NEO Five-Factor Inventory Item 54	0.0185	Short Penn Continuous Performance Test: True Negatives	0.0022
16	Penn Progressive Matrices: Median Reaction Time for Correct Responses	0.0177	NIH Toolbox Meaning and Purpose Survey: Unadjusted Scale Score	0.0019
17	NIH Toolbox Meaning and Purpose Survey: Unadjusted Scale Score	0.0177	NEO Five-Factor Inventory Item 53	0.0017
18	NEO Five-Factor Inventory Item 5	0.0173	Pittsburgh Sleep Quality Index: Minutes to Fall Asleep	0.0017
19	NEO Five-Factor Inventory Item 19	0.0168	Gambling Task: Percentage of Larger Responses in Reward Trials	0.0016
20	Short Penn Continuous Performance Test: Median Response Time for True Positive Responses	0.0167	NIH Toolbox Self-Efficacy Survey: Unadjusted Scale Score	0.0016

Note. Only the 20 highest-ranking predictors are shown for each modelling approach. For the Elastic Net framework, the final selected model had α = 0 and therefore corresponded to ridge regression; predictor importance was summarized using the absolute magnitude of standardized regression coefficients (|β|). For multi-level categorical predictors, such as age, importance was aggregated across the corresponding encoded terms using the Euclidean norm. For XGBoost, predictor importance was quantified in the independent holdout sample using mean absolute SHAP values. Larger values indicate greater contribution to model predictions. These importance measures describe predictive contributions within the fitted models and should not be interpreted as causal effects or independent associations.
